# Quantitative and Selective Surface Plasmon Resonance Response Based on a Reduced Graphene Oxide–Polyamidoamine Nanocomposite for Detection of Dengue Virus E-Proteins

**DOI:** 10.3390/nano10030569

**Published:** 2020-03-21

**Authors:** Nur Alia Sheh Omar, Yap Wing Fen, Jaafar Abdullah, Amir Reza Sadrolhosseini, Yasmin Mustapha Kamil, Nurul ‘Illya Muhamad Fauzi, Hazwani Suhaila Hashim, Mohd Adzir Mahdi

**Affiliations:** 1 Institute of Advanced Technology, Universiti Putra Malaysia, Serdang 43400 UPM, Malaysia; nuralia.upm@gmail.com (N.A.S.O.); amir1348@gmail.com (A.R.S.); 2 Faculty of Science, Universiti Putra Malaysia, Serdang 43400 UPM, Malaysia; jafar@upm.edu.my (J.A.); illyafauzi97@gmail.com (N.I.M.F.); hazwanisuhaila@gmail.com (H.S.H.); 3 inLAZER Dynamics Sdn Bhd, InnoHub Unit, Putra Science Park, Universiti Putra Malaysia, Serdang 43400, Malaysia; yasminmustaphakamil@gmail.com; 4 Wireless and Photonics Network Research Centre, Faculty of Engineering, Universiti Putra Malaysia, Serdang 43400 UPM, Malaysia; mam@upm.edu.my

**Keywords:** dengue virus, envelope proteins, graphene oxide, polyamidoamine, surface plasmon resonance

## Abstract

Dengue viral infection is one of the most common deadliest diseases and has become a recurrent issue for public health in tropical countries. Although the spectrum of clinical diagnosis and treatment have recently been established, the efficient and rapid detection of dengue virus (DENV) during viremia and the early febrile phase is still a great challenge. In this study, a dithiobis (succinimidyl undecanoate, DSU)/amine-functionalized reduced graphene oxide-–polyamidoamine dendrimer (DSU/amine-functionalized rGO–PAMAM) thin film-based surface plasmon resonance (SPR) sensor was developed for the detection of DENV 2 E-proteins. Different concentrations of DENV 2 E-proteins were successfully tested by the developed SPR sensor-based system. The performance of the developed sensor showed increased shift in the SPR angle, narrow full-width–half-maximum of the SPR curve, high detection accuracy, excellent figure of merit and signal-to-noise ratio, good sensitivity values in the range of 0.08–0.5 pM (S = 0.2576°/pM, R^2^ = 0.92), and a high equilibrium association constant (K_A_) of 7.6452 TM^−1^. The developed sensor also showed a sensitive and selective response towards DENV 2 E-proteins compared to DENV 1 E-proteins and ZIKV (Zika virus) E-proteins. Overall, it was concluded that the Au/DSU/amine-functionalized rGO–PAMAM thin film-based SPR sensor has potential to serve as a rapid clinical diagnostic tool for DENV infection.

## 1. Introduction

The demand for rapid, sensitive, and quantifiable methods for the detection of dengue virus is significant. Optical sensors have emerged as a promising technology to potentially identify biological phenomena. They have the advantages of being highly sensitivity, allowing fast detection and real-time measurements, and having a simple configuration [1,2,3,4,5,6,7]. The main advantage of optical sensors is the possibility of performing label-free quantitative detection, which allows direct binding without fluorescent labels or isotope labelling [8,9,10,11,12,13,14]. Recently, two well-known optical sensors have been used to identify and quantify the dengue virus, i.e., the tapered optical fiber (TOF) and the surface plasmon resonance (SPR) sensors. A study using TOF revealed the successful detection of dengue virus (DENV) at 1 pM within 15 min [15]. However, this sensor requires precise alignment, high cleanliness and maintenance, and very careful handling, since the fibers can break easily [16,17]. To address these downsides, researchers are exploring the SPR sensor which has a low cost, is simple to use and environment-friendly, and provides high accuracy and fast measurements [18,19,20,21,22]. However, efficient and highly sensitive SPR sensors are still lacking. Therefore, in this work, we developed a quantitative SPR sensor with a novel biomolecular recognition function and improved sensitivity. Anchoring a stable biorecognition element on the gold surface of the sensor is important to support its antigen binding activity, because it allows a significant change in the angle of the reflectivity minimum and is thus well suited for dengue virus detection.

Graphene oxide (GO)-based sensors have received great attention due to their exciting properties including abundant functional groups, low thickness, very low mass, large specific area, high π-conjugation structure, and high mechanical strength [23,24,25,26,27,28]. Rich functional groups on GO could disturb conductive regions for resistance transduction, and heavily oxidized GO is not electrically conductive [29,30]. By chemical reduction of GO, the oxidized functional groups are removed resulting in reduce graphene oxide (rGO), which is a conductive material. The advantages of rGO with respect to GO are that it can be stored longer without agglomeration, is more stable in organic solvents, and has inferior electrical properties [31,32,33]. Interestingly, some oxygen groups on the rGO surface may be chemically functionalized for the preparation of composite materials. For instance, the functionalization of rGO with primary amines (-NH_2_) renders it hydrophilic and increases its interfacial binding to materials of interest, thus making it more adaptable as a sensing platform for the detection of dengue virus [34,35,36]. Likewise, combining globular-shaped dendrimer of polyamidoamine (PAMAM) with rGO provides great opportunities to enhance the sensitivity of detection. PAMAM dendrimers are believed to provide advantages in sensing applications as they are highly efficient in transporting bioactive agents and are not toxic at the dosages used [37,38,39,40].

With no effective and licensed vaccines, early detection of DENV is crucial to prevent severe clinical complications, which can cause failure of the circulatory system and the liver and death if not adequately managed. There are four distinct serotypes of dengue viruses, i.e., DENV 1 to DENV 4, and each serotype consists 3 structural proteins (capsid, membrane, envelope (E) protein) and 7 non-structural proteins [41,42,43,44,45,46,47]. Currently, the detection of the NS1 antigen from the dengue virus is the basis for early diagnosis and an ideal diagnostic marker of disease progression when antibody levels are not detectable [48,49]. The setback of NS1 detection, however, is its low sensitivity in the diagnosis of secondary dengue infection (DENV 2) compared to primary dengue infection (DENV 1) [50,51,52]; therefore, we did not focus on DENV 2 detection. Additionally, NS1 is a metabolic product that is induced by the virus infection after the viremia phase and therefore allows early detection of DENV, at the onset of infection. With the proposed sensor, our aim was to detect the virus itself through the E-proteins of DENV 2, which was our determinant. The E-proteins are the major virion surface proteins and seem to be a promising target since they contribute to the formation of the coat of the virus itself and therefore it are sufficient to mount an early immune response (in the viremia phase) [53].

Herein, we developed an SPR sensor based on an amine-functionalized rGO–PAMAM composite, with monoclonal antibodies immobilized on self-assembled dithiobis (succinimidyl undecanoate, DSU) to detect and quantify the dengue virus. The sensing performances of the proposed sensor, i.e., linear sensitivity, detection accuracy, full-width half maximum, signal-to-noise ratio, figure of merit, selectivity, and matrix effect are discussed in detail. To the best of our knowledge, this is the first report of the detection of DENV at the low concentration of 0.08 pM in 8 min using an Au/DSU/amine-functionalized rGO–PAMAM/IgM thin film-based SPR optical sensor.

## 2. Materials and Methods

### 2.1. Reagents

DSU (MW = 628.84 g/mol) was purchased from Dojindo Japan. Graphene oxide was purchased from Graphanea, Spain. PAMAM dendrimer (ethylenediamine core, generation 4.0 solution in methanol), ethylenediamine (EDA), N-hydroxysuccinimide (NHS), bovine serum albumin (BSA) were purchased from Sigma Aldrich, Germany. *N*-Ethyl-*N*-(3-(dimethylaminopropyl) carbodiimide (EDC) was bought from Fluka, Switzerland. The standard powder of recombinant DENV 2 E-proteins and antibodies (IgM) against dengue type 2 envelope proteins were purchased from Meridian Life Science. The preparation of amine-functionalized rGO was begun by amalgamating the graphene oxide with EDC for 5 min, followed by the addition of EDA. After the solution was vigorously stirred, the suspension was slowly dissolved and started to change its color from brown to dark black. Then, the suspension was washed for 5 times with ethanol and centrifuged to remove excessive EDA and EDC and then was allowed to dry in an oven at 60 °C for at least 1 h. Afterward, the PAMAM solution with the desired concentration was mixed to the amine-functionalized rGO to obtain a composite. Unless otherwise indicated, all antibodies and antigen solutions were diluted in 10 mM phosphate-buffered saline (PBS) at pH 7.4. All chemicals were of reagent or higher grade, and deionized water was used throughout the experiments.

### 2.2. Sensor Surface Fabrication

To produce the best SPR peak for sensor application, a metal deposition technique to obtain a thin Au film (~50 nm) with high thickness tolerance is required. The generally used methods for metal thin-film deposition are electroplating, sputtering, and evaporation (such as vacuum thermal, electron beam, laser beam, and ion-plating evaporation) [54,55,56]. Sputter coating was chosen in this study because it achieves uniform thickness and high film quality, meeting the thickness tolerance requirement for the SPR experiment. It is also an easy-to-use, environment-friendly, and relatively low-cost technique.

**Figure 1:**
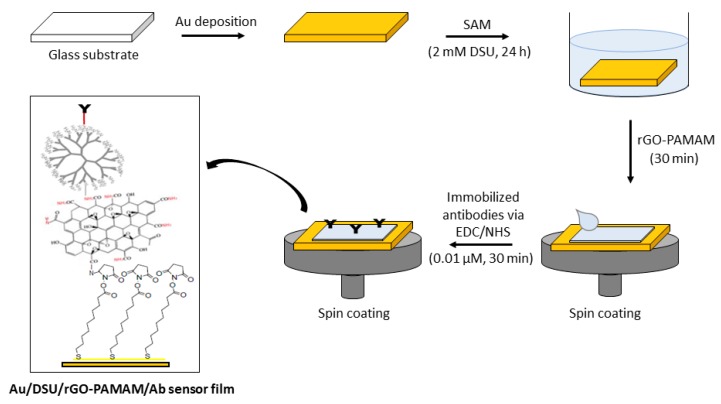
Preparation procedure of the proposed sensor film. SAM: self-assembled monolayer, EDC: *N*-ethyl-*N*-(3-(dimethylaminopropyl) carbodiimide, NHS: *N*-hydroxysuccinimide, DSU/rGO–PAMAM: dithiobis (succinimidyl undecanoate)/ reduce graphene oxide–polyamidoamine.

Figure 1 shows the preparation procedure of the proposed sensor film. To begin, a glass substrate (Menzel glass, 24 mm × 24 mm) was coated with an Au film (~50 nm) using a sputter coater, with a deposition time of 67 s and a current of 20 mA. The prepared Au film was then rinsed thoroughly using deionized water and then ethanol and dried in a nitrogen flow. Afterward, the Au film was self-assembled in 2 mM DSU solution for 24 h to allow chemisorption of biomolecules through amide linkages. After rinsing with acetone and PBS, the gold substrate was incubated with amine-functionalized rGO–PAMAM for 30 min, followed by spinning. The amine-functionalized rGO–PAMAM-modified film was cross-linked with EDC/NHS for 30 min and spun at 6000 rpm for 30 s. After cross-linking, 0.01 µM antibodies specific to DENV 2 E-proteins was incubated over the surface for 30 min. This sensor film will be referred to as DSU/amine-functionalized rGO–PAMAM/IgM in the following discussion.

### 2.3. DENV E-Proteins Detection

**Figure 2:**
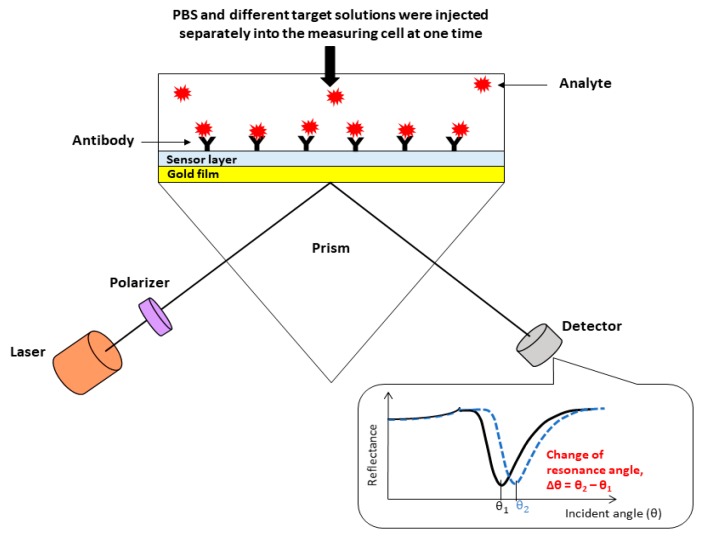
Schematic of the custom-made surface plasmon resonance (SPR) set up (with the illustration of the SPR curve and the change of the resonance angle).

For the detection of DENV 2 E-proteins, the sensor film was mounted on a prism with a refractive-index-matching liquid and loaded onto the SPR rotating stage for SPR measurements. Figure 2 shows the schematic diagram of our custom-made SPR experimental set up. In the beginning, 1 mL of PBS solution was injected into the measuring cell to obtain the baseline signal. Afterward, 1 mL of 0.08 pM DENV 2 E-proteins solution was injected into the measuring cell while monitoring the SPR response for 30 min. A triplicate SPR response was recorded for each concentration of DENV 2 E-proteins solution (0.08–1 pM) by using new sensor films. The changes of the SPR shift for each concentration of DENV 2 E-proteins were calculated by measuring the difference between the resonance angle of the sample and the PBS solution (as shown in Figure 2). The selectivity of the proposed sensor was also evaluated by comparing its SPR response to different concentrations of DENV 2 E-proteins with that to other proteins.

### 2.4. SPR Performances

There are several parameters, i.e., sensitivity, full width at half minimum (FWHM), detection accuracy (DA), signal-to-noise ratio (SNR), figure of merit (FOM), selectivity, and spike recovery that are used to analyze the performance of SPR sensors. The sensitivity of an SPR sensor can be defined by calculating the slope in the graph of the resonance angle shift, Δ*θ*, versus DENV 2 E-protein concentrations, as follows:

(1) $$S=\frac{\Delta \theta }{\left[DENV\right]}$$

A second parameter is the angular width of the SPR curve for the half value of the maximum reflectance, which is known as the FWH). The value of FWHM should be as small as possible in order to get an accurate reading on the dip of the SPR curve [57]. Therefore, the DA of the SPR sensor is y inversely proportional to the value of FWHM.

To comprehensively evaluate the performance of the SPR signal, the SNR can be determined according to

(2) $$SNR=\frac{\Delta \theta }{FWHM}$$

Finally, the FOM can be defined as the ratio between the spectral sensitivity and the width of the SPR curve and can be written as

(3) $$FOM=\frac{S}{FWHM}$$

### 2.5. Sensor Surface Characterization

The surface morphology and roughness of the sensor films were imaged using an atomic force microscope (AFM, Bruker AFM multimode 8, Santa Barbara, CA, USA) in Scan Asyst mode. This measurement was performed in ambient atmosphere at room temperature.

## 3. Results

**Figure 3:**
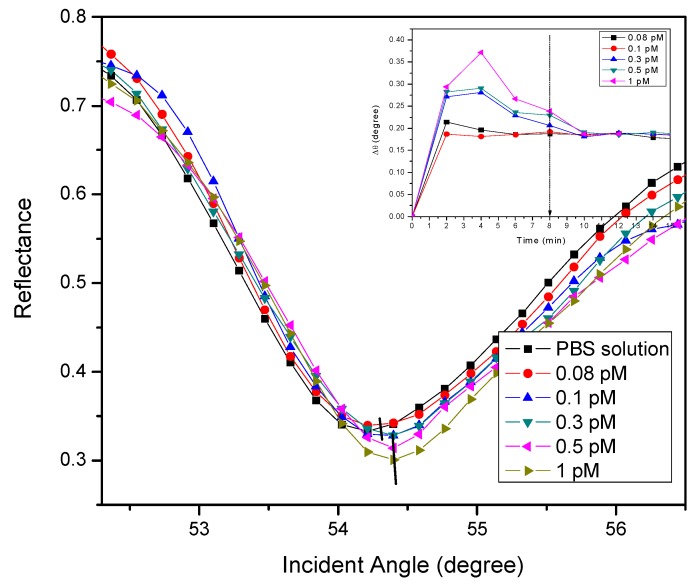
SPR reflectance of the SPR sensor based on a DSU/amine-functionalized rGO–PAMAM/IgM sensor film. Inset: SPR time response upon introduction of 0.08 to 1 pM of DENV 2 E-proteins.

Figure 3 shows the SPR responses of the proposed sensor film for the detection of DENV 2 E-proteins. To verify the viability of the sensor film, various concentrations of DENV 2 E-proteins in the range of 0.08-1 pM were injected into the cell. The results showed that the resonance angle for the reference solution (0 pM), i.e., the PBS solution, was 54.2138°. When the proposed sensor was exposed to 0.08 pM DENV 2 E-proteins, the resonance angle of the reflected light increased to 54.3052°. Subsequently, for DENV 2 E-proteins concentrations of 0.1 pM, 0.3 pM, 0.5 pM, and 1 pM, the resonance angles from the SPR curves were found to be 54.3137°, 54.3925°, 54.4004°, and 54.4083°, respectively. To measure the amount of antigen bound to the sensor surface, the resonance angle shift (Δ*θ*) was determined from the difference between the resonance angle of the antigen and the resonance angle of the reference solution. It was found that increases of Δ*θ* of 0.0914°, 0.0999°, 0.1708°, 0.1866°, and 0.1945° were obtained when detecting 0.08 pM, 0.1 pM, 0.3 pM, 0.5 pM, and 1 pM DENV 2 E-proteins, respectively. These Δ*θ* can be attributed to the changes in the refractive index of the sensor surface which in turn changed the real part of the dielectric constant of the gold film caused by the binding of DENV 2 E-proteins. It was inferred that a change of the thickness of the sensing layer would also result in a slight angle shift of SPR, as the evanescent wave possesses longer penetration depths [58,59]. The inset of Figure 3 shows how Δ*θ* varied with time when detecting different concentrations of DENV 2 E-proteins. It can be seen that the proposed sensor achieved a stable detection time of 8 min for the lowest concentration of DENV 2 E-proteins. Therefore, all DENV E-proteins concentrations were left for 8 min before the SPR curve was recorded.

**Figure 4:**
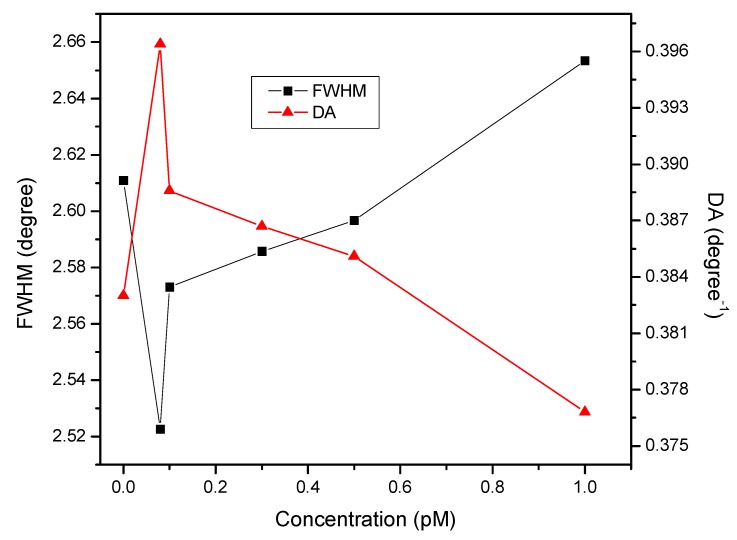
Full width at half minimum (FWHM) and detection accuracy (DA) of the SPR sensor based on a DSU/amine-functionalized rGO–PAMAM/IgM sensor film.

Figure 4 reports the evolution of FWHM and DA as a function of the concentration of DENV 2 E-proteins. As expected, the most extensive broadening of the SPR curves was observed when the proposed sensor surface was exposed to the highest concentration of DENV 2 E-proteins. This phenomenon was due to the increasing refractive index on the sensor surface caused by the kinetics of the binding between ligand and analyte [60]. The increase in surface roughness also led to broadening of the SPR curves as the coupling rate of SPR became poor. On the other hand, the resolution of the SPR signal decreased due to the increase in electron energy loss in the dense sensing layer. The minimum value of FWHM was achieved with the first introduction of DENV 2 E-proteins at the concentration of 0.08 pM. A more distinct and narrower SPR curve allows for higher accuracy of SPR sensors throughout measurements. Thus, the highest DA of 0.3964 degree*^−^*^1^ was achieved in the detection of 0.00008 nM DENV E-proteins.

**Figure 5:**
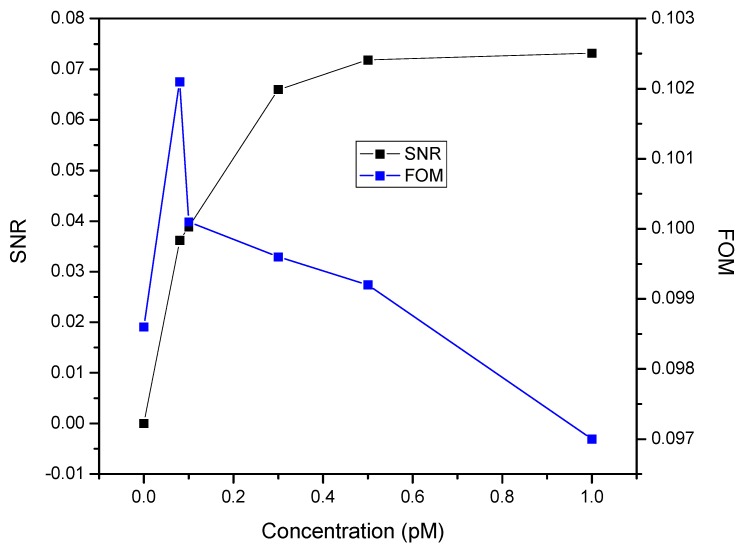
Signal-to-noise ratio (SNR) and figure of merit (FOM) of the SPR sensor based on a DSU/amine-functionalized rGO–PAMAM/IgM sensor film.

Figure 5 depicts the variation of SNR and FOM with the concentration of DENV 2 E-proteins. Expectedly, the SNR increased with the increase of the concentration of DENV 2 E-proteins. This indicated that a high concentration of DENV 2 E-proteins led to a lower noise of the signal, thus providing the maximum values of SNR. This effect was due to the stronger coupling of surface plasmon, which led to a stronger radiation damping of the surface [61,62]. In addition, the variation in SNR was almost similar to the binding kinetic analysis, as it is dependent on the resonance angle shift. Beyond that, a high FOM is required for a better quality of the SPR sensor. Apparently, a higher FOM was observed when a 0.08 pM DENV 2 E-proteins solution was introduced. The reason for the higher value of FOM at this concentration could be the smaller dissipation in metal upon the excitation of surface plasmon at a shorter wavelength, resulting in a narrow full width at half maximum of the SPR curve. Consequently, the FOM decreased with a further increase of the concentration of DENV 2 E-proteins . The decreasing of the FOM could be due to a faster decrease in the sensitivity compared to FWHM, which resulted in a notable uncertainty in determining the position of the resonance angle.

**Figure 6:**
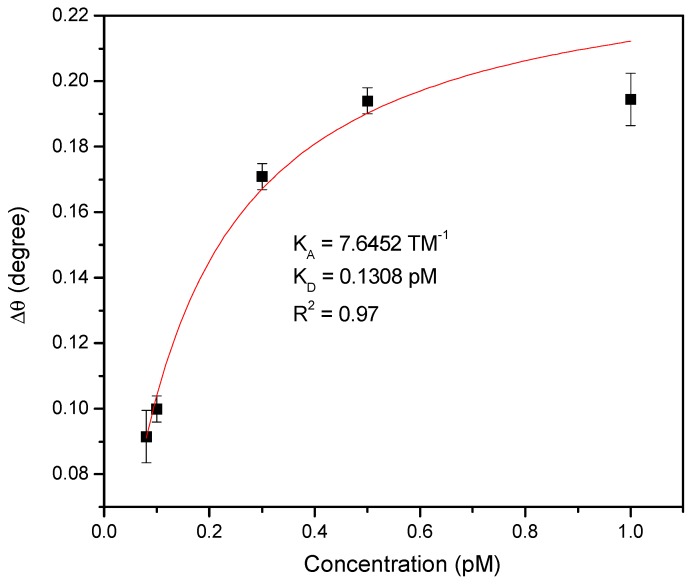
Binding affinity between the Au/DSU/amine-functionalized rGO–PAMAM/IgM sensor film and secondary dengue virus infection (DENV 2) envelope (E)-proteins depending on the concentration of the latter.

To investigate the binding between Au/DSU/amine-functionalized rGO–PAMAM/IgM-based SPR sensor and target, the changes in the resonance angle as a function of the concentration of DENV 2 E-proteins from 0.08 to 1 pM were plotted and fitted according to the Langmuir adsorption model (Figure 6), represented by the following equation [63,64]:

(4) $$\Delta \theta =\frac{\Delta \theta_{\max}C}{K_{D}+C}$$

where Δ*θ*_max_ is the maximum SPR shift at saturation, *C* is the concentration of DENV, and *K_D_* is the equilibrium dissociation constant. Typically, the strength of biomolecular interactions is quantified through *K_D_* (reciprocal of *K_A_*). The fitted graph gave a *K_D_* of 0.1308 pM with R^2^ of 0.97, which was found to be consistent with the standard *K_D_* value for protein interactions (*K_D_* < 10 nM) [65,66]. A smaller *K_D_* value led to higher binding affinity of the ligand for its target. The apparent *K_A_* values for the binding of DENV 2 E-proteins to the sensor surface was 7.6452 TM^−1^, indicating the high potential for the use of the proposed SPR sensor in detecting DENV 2 E-proteins.

**Figure 7:**
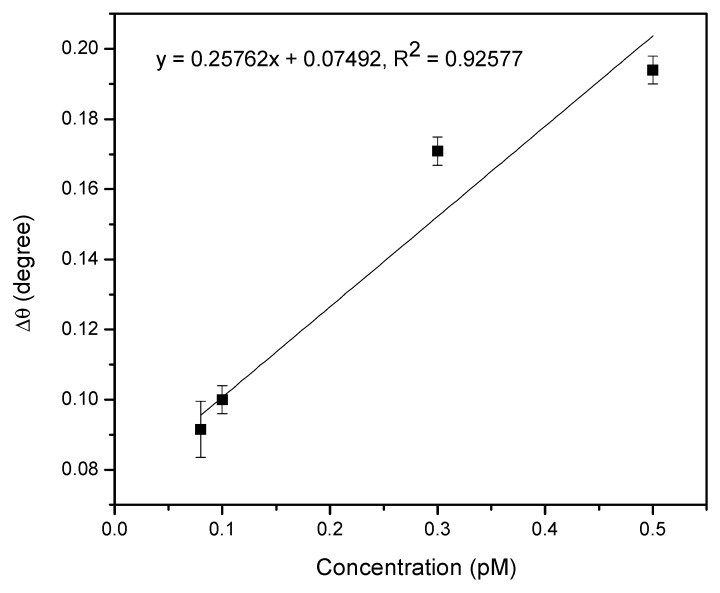
Relationship between Δ*θ* and different concentrations of DENV 2 E-proteins.

In order to determine the sensitivity of the proposed sensor film for DENV 2 E-proteins, a linear correlation curve was plotted and is presented in Figure 7. The results were fitted linearly with a correlation coefficient, R^2^, of 0.92. The slope of the linear fit was considered to be the sensitivity value of the proposed SPR sensor, thus yielding a sensitivity of 0.2576°/pM. It appears that DENV 2 E-proteins can be sensitively detected at the lowest concentration of 0.08 pM. This is possible thanks to the great penetration depth of the SPR evanescent field along the DSU/amine-functionalized rGO–PAMAM/IgM sensor layer.

Table 1 presents the performance of other techniques developed for DENV detection, in terms of sensitivity and detection limit [67,68,69,70,71]. From the table, it can be seen that the sensitivity of these techniques has not been extensively evaluated compared to their detection limit. Therefore, with respect to the detection limit, the lowest DENV concentration measured by our SPR sensor was 0.08 pM. The performance of our SPR sensor is highly dependent on its surface modification, which can cause significant changes in surface mass due to binding events, thus improving its sensing performance.

**Figure 8:**
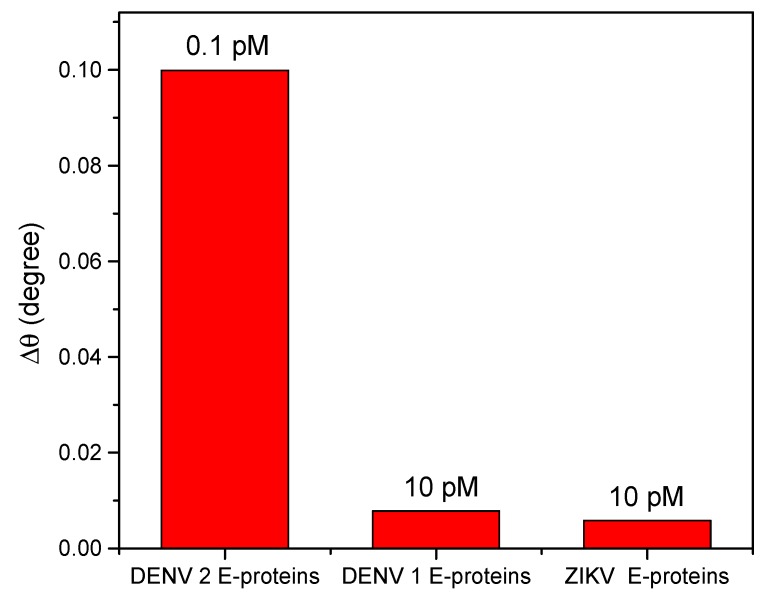
Shift of the resonance angle measured with different antigens.

To determine the selectivity of the Au/DSU/amine-functionalized rGO–PAMAM/IgM sensor film towards DENV 2 E-proteins, other envelope proteins, such as DENV 1 E-proteins and ZIKV E-proteins, at the concentration of 10 pM were tested. An excellent sensor should be highly selective in the detection of DENV 2 E-proteins. The results are shown in Figure 8. Clearly, the highest SPR signal was obtained for the detection of DENV 2 E-proteins, even of their concentration was much lower than those of DENV 1 E-proteins and ZIKV E-proteins. This demonstrated that the proposed sensor is highly selective for the detection of DENV 2 E-proteins.

**Figure 9:**
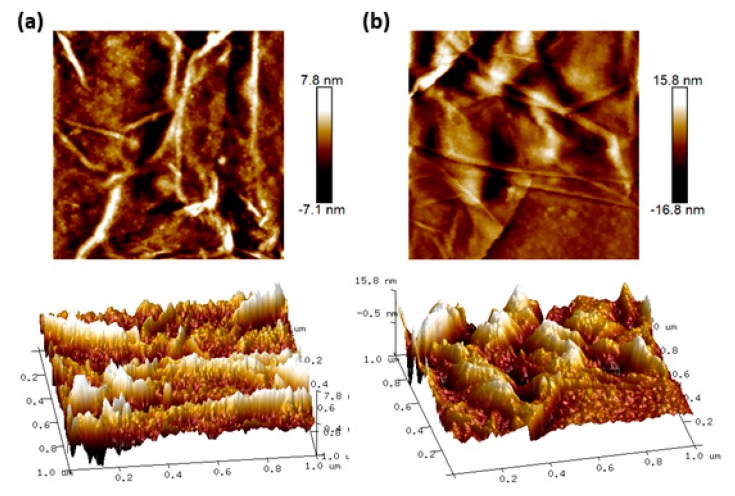
AFM images of the Au/DSU/amine-functionalized rGO–PAMAM/IgM sensor film (**a**) before and (**b**) after the introduction of DENV 2 E-proteins.

AFM images were taken before (Figure 9a) and after the introduction of DENV 2 E-proteins (Figure 9b) onto the surface of the Au/DSU/amine-functionalized rGO–PAMAM/IgM sensor. A crumpled and wrinkled morphology of the rGO–PAMAM structure is observed in both images. It can be observed that when DENV 2 E-proteins were present onto the sensor surface, the surface structure became less crumpled and wrinkled. The surface roughness of the sensor films increased from 1.56 to 3.19 nm upon the introduction of DENV 2 E-proteins. The change in surface roughness proves that the attachment of DENV 2 E-proteins changed the surface morphology of the sensing layer.

## 4. Conclusions

In this study, a DSU/amine-functionalized rGO–PAMAM thin film-based SPR sensor was developed for the detection and quantification of DENV 2 E-proteins. The results demonstrate that the developed sensor could successfully monitor the changes in the SPR angle when detecting the lowest concentration of DENV 2 E-proteins, i.e., 0.08 pM, within 8 min. The sensitivity and binding affinity constant of the developed sensor were 0.2576°/pM and 7.6452 TM^−1.^ The highest FOM and DA values were obtained when detecting 0.08 pM of DENV 2 E-proteins and corresponded to the minimum values of SNR. The selectivity of the proposed sensor was demonstrated, since the SPR response to other proteins was reduced compared to that to DENV 2 E-proteins. It can be concluded that the developed SPR sensor has a great potentiality for rapid DENV diagnostic analyses or point-of-care tests.

## Figures and Tables

**Table 1:** Performance of techniques developed for DENV (dengue virus) detection.

Technique	Target	Sensitivity	Limit of Detection	Reference
ELISA	NS1	-	0.02 nM	67
LSPR	NS1	43 nm/(ng/mm^2^)	1.54 nM	68
Rapid kits	NS1	-	0.1 nM	49
Electronic biosensor	E-proteins	-	2.11 pM	69
Tapered fiber sensor	E-proteins	5.02 nm/nM	1 pM	70
SPR Biacore 3000 (automated)	IgM antibodies	0.0132 pM/sec.^−1^	2.125 pM	71
Modified SPR sensor (custom-made)	E-proteins	0.2576°/pM	0.08 pM	This work
